# Prehospital undertriage of older injured patients in western Switzerland: an observational cross-sectional study

**DOI:** 10.1186/s13049-024-01271-5

**Published:** 2024-10-08

**Authors:** Clément Poncet, Pierre-Nicolas Carron, Vincent Darioli, Tobias Zingg, Francois-Xavier Ageron

**Affiliations:** 1 School of Medicine, University of Lausanne, Lausanne, Switzerland; 2 Department of Emergency Medicine, Lausanne University Hospital and University of Lausanne, Bugnon 46, 1011 Lausanne, Switzerland; 3 Department of Visceral Surgery, Lausanne University Hospital and University of Lausanne, Lausanne, Switzerland

**Keywords:** Prehospital, Injuries, Trauma, Frail elderly, Triage, Older adults, Aged

## Abstract

**Background:**

The ageing of the population is leading to an increase in the number of traumatic injuries and represents a major challenge for the future. Falls represent the leading cause of Emergency department admission in older people, with injuries ranging from minor to severe multiple injuries. Older injured patients are more likely to be undertriaged than younger patients. The aim of this study was to investigate the extent of undertriage in older patients with particular emphasis on access to trauma centres and resuscitation rooms.

**Methods:**

Retrospective observational cross-sectional study based on data prospectively collected from prehospital electronic records including all patients ≥ 18 years for whom an ambulance or helicopter was dispatched between 1 January 2018 and 31 April 2023 due to a trauma. The primary outcome, admission to the resuscitation room of the regional trauma centre with trauma team activation, was assessed by age. Multivariate logistic regression was used to control for known confounders and to test for plausible effect modifiers.

**Results:**

Emergency Medical Services treated 37,906 injured patients. Older patients ≥ 75 years represented 17,719 patients (47%). Admission to trauma centre with trauma team activation was lower in older patients, N = 121 (1%) compared to N = 599 (5%) in younger patients, *p* < 0.001; adjusted odds ratio: 0.33 (0.24–0.45); *p* < 0.001. Undertriage increased by twofold with age ≥ 75; OR: 1.81 (1.04–3.15); *p* value < 0.001. Undertriaged patients were older, more likely to be female, to have low energy trauma and to be located farther from the regional trauma centre.

**Conclusion:**

Older injured patients were at increased risk of undertriage and non-trauma team activation admission, especially if they were older, female, had head injury without altered consciousness and greater distance to regional trauma centre.

**Supplementary Information:**

The online version contains supplementary material available at 10.1186/s13049-024-01271-5.

## Background

The ageing of the world’s population, particularly in Europe, is an increasingly important demographic trend with projections showing a significant increase in the proportion of people over 65 (20.3% over 65 in 2020 and 29.4% in 2050) [1]. Switzerland reflects this trend, with an increasing proportion of older people. Falls represent the leading cause of Emergency department admission among older people. In addition to this major public health issue, older people are becoming involved in activities that used to be reserved for younger people such as driving, mountaineering and extreme sports due to longer life expectancy and improved health [2, 3]. Trauma in older people is a challenge for the years to come.

Despite advances in medical care, studies consistently show that older people have a worse prognosis than their younger counterparts following injuries of similar severity [4]. This discrepancy in outcomes can be attributed to factors such as reduced physiological resilience due to ageing, and potential underestimation of injury severity by paramedics or healthcare professionals, leading to suboptimal care.

In addition, older injured patients often present with unique challenges, including altered pain perception, cognitive impairment and multiple comorbidities, often associated with medication use, including anticoagulants, β-blockers or antiplatelet agents. These factors can exacerbate their condition and complicate clinical assessment and management, making it imperative to recognise and address the specific needs of this population.

Evidence suggests that older injured patients are more likely to be undertriaged than younger patients with similar Injury Severity Scores (ISS), particularly when accessing critical care facilities such as trauma centres and resuscitation units [5]. Undertriage refers to the situation where a patient has severe injury is considered as less severely injured [6]. Undertriage might have harmful consequences, leading to increased morbidity and mortality in this vulnerable population.

Considering these concerns, the present study aims to investigate the extent of undertriage of older injured patients in the canton of Vaud, with a particular focus on access to trauma centres and resuscitation rooms. By identifying and addressing gaps in triage protocols and healthcare delivery, this research aims to improve outcomes and quality of care for this growing population.

## Methods

### Design of the study

We performed a retrospective observational cross-sectional study based on anonymised public health data collected prospectively from prehospital electronic records in the canton of Vaud.

### Patients and data collection

We included patients ≥ 18 years old for whom an ambulance or helicopter was dispatched between 1 January 2018 and 31 April 2023 consecutive to a trauma. We excluded patients < 18 years old and without significant injury, defined by the National Advisory Committee for Aeronautics (NACA) scale of 0 and 1. We excluded patients with a NACA of 7 who died at the scene because they were not transported to hospital. As we expected undertriage of older patients, we included all patients for whom an ambulance was dispatched and not only those suspected of major trauma based on initial assessment or retrospectively by the Injury Severity Score (ISS).

### Setting

The Emergency Medical Service (EMS) of the canton of Vaud is a two-tier system in Switzerland covering more than 800,000 inhabitants. In the event of a trauma, the dispatch centre alerts the nearest ambulance. If the dispatch centre or the ambulance in the field identifies severity criteria, a mobile intensive care unit (MICU) staffed by a prehospital emergency physician is dispatched by ground ambulance or by helicopter. Severity criteria include compromised airway or breathing, haemodynamic instability, unconsciousness, spinal cord injury resulting in paralysis, penetrating injuries, limb amputation, incidents involving vehicle ejection, the need for extrication or scenarios involving more than three injured people.

There are 14 ambulance bases in the canton (between 23 and 29 ambulances can be dispatched simultaneously according to time of day), 6 MICUs with ambulances staffed by emergency physicians, a Helicopter Emergency Medical Service (HEMS). The canton of Vaud has seven general hospital and one university hospital which is both the regional trauma centre and a local hospital.

### Outcomes

The primary outcome is admission to the resuscitation room (RR) of the regional trauma centre with trauma team activation (TTA).

We expected that older patients would have less care and less access to trauma centres than younger people. Undertriage could also be observed with less dispatched MICU and Helicopter Emergency Medical Service (HEMS). Secondary outcomes are admission to trauma centre, MICU or HEMS dispatched, prehospital interventions such as intubation, analgesia, tranexamic acid and vasoactives drugs.

### Exposure

We assessed outcomes by age group (< 55 years, 55–74; ≥ 75 years) and by age as a continuous variable.

As we expected that admission to RR with TTA would vary with the presence of major trauma, we estimated the risk of severity using validated prognostic models. Prehospital triage refers to the clinical assessment of the probability of life-threatening injury based on vital signs, level of consciousness, anatomical criteria and mechanism of injury. To estimate which patient should have been transported to the regional trauma centre, we estimated the risk of major trauma using validated prognostic model including these criteria available at the site of the accident. The BATT score estimates the risk of haemorrhage by predicting traumatic death from bleeding and early death [7]. The MGAP score estimates the risk of major trauma by predicting traumatic death at 28 days [8]. The BATT score has been externally validated in the UK and Switzerland and predicts the risk of death from bleeding and early death [7, 9]. We performed adjusted analysis using the BATT score and the MGAP. We did not include the Injury Severity Score (ISS) as a potential confounder because it was not estimated in the prehospital database. Moreover, the ISS is not useful for prehospital triage because it cannot be estimated in the prehospital setting and because less severe injuries can be life-threatening for older patient. The ISS is a criterion for retrospective evaluation of the triage protocol but not for deciding which patient should be transported to the trauma centre.

### Statistical analysis

We first described the characteristics of the injured patients by age and compared the proportions using the Chi-2 test. Continuous variables were expressed as mean and SD or median and interquartile range, depending on the distribution. Student t-test was used to compare the means of continuous variables.

We examined the difference in prehospital interventions by age categories. We used interaction tests (Chi-squared test of homogeneity) to see whether the prehospital interventions differed by age.

As most older injured patients have a low risk of major trauma and don’t meet the criteria for full trauma team activation, we expected an interaction with the BATT and the MGAP scores, which estimate the risk of major trauma at baseline. We will examine the effect of undertriage in the different categories of baseline risk. We defined different risk categories for the BATT score: risk unlikely: BATT score < 3 (mean death from bleeding < 0.5%); Low risk: BATT score 3 to 4 (< 1%); Intermediate risk: BATT score 5 to 7 (1 to 2%); High risk: BATT score ≥ 8 (10%) and for the MGAP score: low risk: MGAP 23 to 29; intermediate risk: MGAP 18 to 22; high risk: MGAP 3 to 17.

We estimated crude odds ratio by category and test the homogeneity of the association with age across categories using CHI-2 test.

**Fig. 1:**
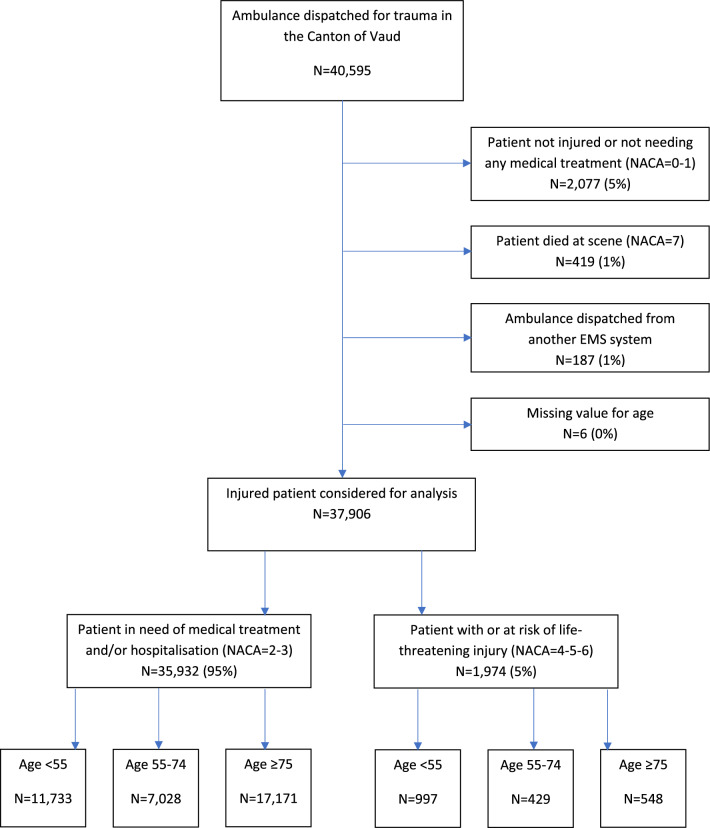
Flowchart of the study population

We explored the relationship between age and outcome by estimating odds ratios and 95% confidence intervals adjusted with potential confounders: sex, BATT score, MGAP score, mechanism of injury (MOI), systolic blood pressure, Glasgow Coma Scale (GCS) and distance from the accident to the trauma centre. We selected covariates in a parsimonious manner, using a Direct Acyclic Graph (supplement files, Figure 1). We then developed different models to test plausible interaction: age and sex, age and baseline risk, age and MOI, age and NACA, age and BATT score, age and High energy trauma, age and penetrating trauma, age and distance to the trauma centre. Continuous variables were included in the model with their linear and polynomial terms. We fitted a multivariate model including confounders and interaction variable. As we also expected an interaction between sex and undertriage, we explored if the undertriage varied by sex.

Finally, we examined the characteristics of undertriaged older patients. As the criteria for RR admission and TTA were not collected in the database, we considered a patient with a high risk of major trauma who was not admitted in the RR to be undertriaged. We defined a high risk of major trauma as a BATT score ≥ 8 and/or a MGAP score < 18. We compared these patients with patients with the same criteria who were admitted to the RR with TTA.

All analyses were performed using STATA software (version 18.0; Stata Corp., College Station, TX, USA).

This study followed the Strobe guidelines.

### Missing data

Age was missing for 6 patients (0.1%). We did not report missing value for the primary outcome. The detail of missing value for each variable are summarized in the Table 1. We performed multiple imputation with chained equation for physiological parameters. We imputed 20 datasets.

**Table 1:** Characteristics of injured patients

	Missing value (%)	Age < 55 years N = 12,730 N (%)	Age 55–74 years N = 7,457 N (%)	Age ≥ 75 years N = 17,719 N (%)
Gender female (%)	0	4,461 (35)	3,689 (49)	12,303 (69)
Mechanism of injury	56 (0)			
Motor vehicle accident		5,281 (42)	1,883 (25)	1,172 (7)
Included High energy		1,059 (8)	321 (4)	147 (1)
Fire gun		22 (0)	8 (0)	30 (0)
Stabbing		341 (3)	38 (1)	35 (0)
Blunt force trauma/crush injury		766 (6)	102 (1)	49 (0)
Fall – low energy and unknown		3,238 (25)	4,226 (57)	14,414 (81)
Fall – high energy		506 (4)	343 (5)	492 (3)
Others		2,587 (20)	861 (12)	1,529 (9)
Transport	12 (0)			
Ambulance		11,901 (94)	7,133 (96)	17,444 (99)
Helicopter		419 (3)	170 (2)	37 (0)
No transport		403 (3)	154 (2)	233 (1)
Distance to regional trauma center	525 (1)	17 (14)	18 (14)	16 (13)
Helicopter, mean Km (sd)		33 (9)	35 (9)	31 (9)
Ambulance, mean Km (sd)		17 (14)	18 (13)	16 (13)
Hospital catchment area outside regional trauma centre		6,850 (56)	4,452 (60)	10,110 (57)
Systolic blood pressure, mean (sd)	4,267 (11)	121 (21)	132 (26)	142 (27)
SBP < 100 mmHg		1,057 (10)	590 (9)	730 (5)
Glasgow coma scale	3,143 (8)			
3–8		217 (2)	80 (1)	100 (1)
9–12		145 (1)	71 (1)	176 (1)
13–15		11,110 (97)	6,746 (98)	16,118 (98)
BATT score	5,992 (16)			
Risk of death unlikely (0–2)		8,641 (85)	5,377 (84)	12,840 (83)
Low risk (3–4)		336 (3)	384 (6)	1,587 (10)
Intermediate risk (5–7)		932 (9)	509 (8)	763 (5)
High risk (≥ 8)		194 (2)	131 (2)	220 (2)
MGAP score	3,143 (9)			
High risk (3–17)		164 (1)	88 (1)	121 (1)
Intermediate risk (18–22)		119 (1)	1,563 (23)	3,472 (21)
Low risk (23–29)		11,189 (98)	5,246 (76)	12,801 (78)

Sd: Standard deviation; SBP: Systolic Blood Pressure; BATT: Bleeding Audit Triage for Trauma; MGAP: Mechanism, Glasgow, Arterial Pressure, Penetrating

## Ethics approval

According to Swiss Ethics, studies using anonymised data are exempt from an informed consent and benefit from a waiver to ethics committee. The database was approved by Commission for Research Ethics on Human Subjects of the canton of Vaud (CER-VD number: 2020–01190). Data provided with permission from the public health service of the Canton of Vaud.

## Results

**Fig. 2:**
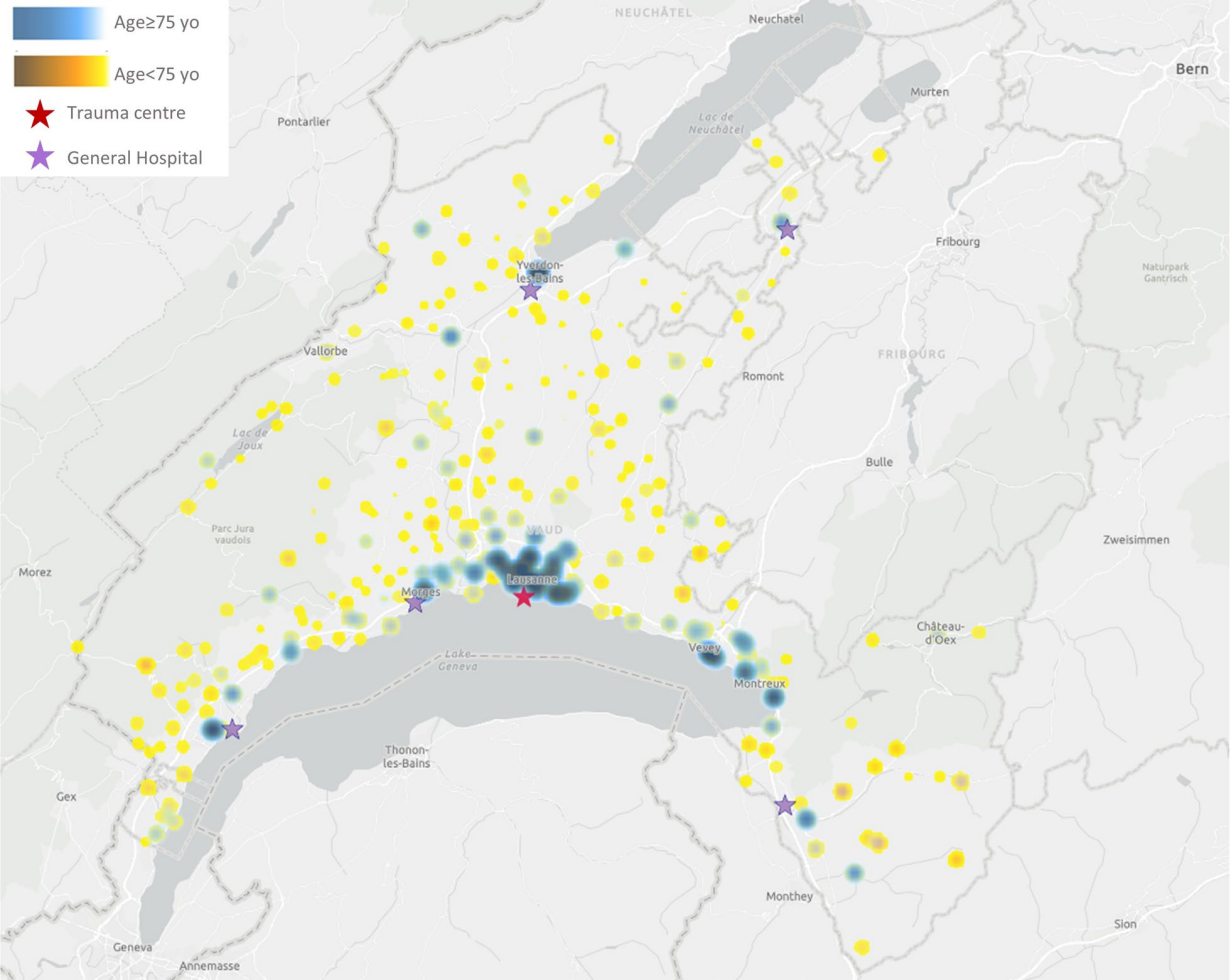
Geospatial analysis by age categories

Between 1st January 2018 and 31 April 2023, 37,906 patients were treated by emergency services in the canton of Vaud and fulfilled the inclusion criteria (Fig. 1). Age was missing for 6 patients. Older patients ≥ 75 years represented 17,719 patients (47%) (Table 1). There were 12,303 older women (69%). High-energy motor vehicle accidents were more common in younger than in older patients, 42% versus 7%, *p* value < 0.001. Falls were predominant in older patients, n = 14,414 (81%). Older patients were less likely to be transported by helicopter than youngers patients, n = 43 (0.2%) versus n = 662 (3.2%); *p* value < 0.001. Mean systolic blood pressure was higher in older than in younger patients, 142 (27) versus 125 (22); *p* value < 0.001. In terms of the baseline risk of significant bleeding, older patients had a low risk of death from bleeding according to the BATT score and most of the younger patients had an intermediate to high risk. The distance from the accident to the trauma centre was slightly lower in older patients, 16 km versus 17 km, *p* value < 0.001. The spatial analysis showed that the location of the accident for older patients was mainly in the area of the regional trauma centre (Fig. 2).

**Fig. 3:**
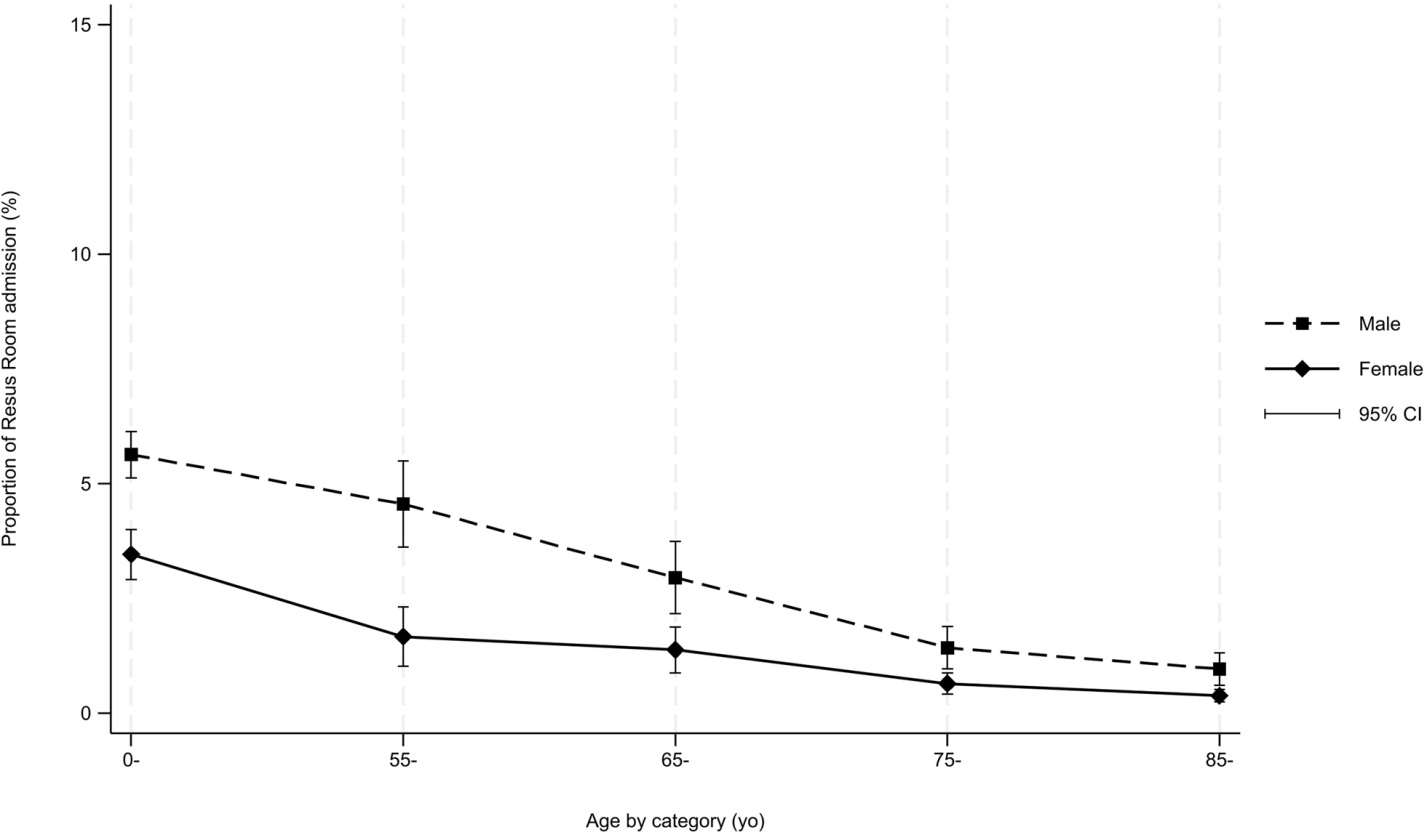
Admission to resuscitation room with trauma team activation by age and gender

Transport to the regional trauma centre and admission to the RR with TTA was lower in older patients (Table 2, Fig. 3). A prehospital MICU in addition to the paramedic was less likely to be dispatched for older patients, n = 457 (3%) versus 1,517 (12%), *p* < 0.001. Older patients received less morphine and fentanyl, n = 4,319 (24%) versus n = 4,530 (36%); *p* value < 0.001 and less tranexamic acid, n = 303 (2%) versus n = 64 (0%); *p* value < 0.001. They are also less likely to be intubated, n = 99 (1%) those < 55 years old versus > 75 years n = 31 (0%); *p* value < 0.001. In univariate analysis, admission to the RR with TTA decreased with age and gender (Fig. 3).

**Table 2:** Outcomes and Therapeutic interventions

	Missing value (%)	Age < 55 years N = 12,730 N (%)	Age 55–74 years N = 7,457 N (%)	Age ≥ 75 years N = 17,719 N (%)	*p* Value
Admission to resuscitation room with TTA (on patient transported N = 37,116)	64 (0)	599 (5)	193 (3)	121 (1)	< 0.001
Admission to regional trauma centre (on patient transported N = 37,116)	4 (0)	5,735 (47)	2,732 (37)	6,384 (37)	< 0.001
Prehospital medical team: HEMS or MICU	0	1,517 (12)	611 (8)	457 (3)	< 0.001
Intubation	–	99 (1)	36 (0)	31 (0)	< 0.001
Morphine or Fentanyl	–	4,530 (36)	2,411 (32)	4,319 (24)	< 0.001
Tranexamic acid	–	303 (2)	101 (1)	64 (0)	< 0.001
Vasoactive drugs	–	90 (1)	32 (0)	32 (0)	< 0.001

TTA: Trauma team activation; HEMS: Helicopter Emergency Medical Service; MICU: Mobile Intensive Care Unit

Age ≥ 75 years was associated with a 70% reduction in the odds of admission to RR with TTA after adjusted for confounders, OR: 0.33 (0.24–0.45); *p* value < 0.001 (Table 3). Undertriage increased by twofold with age ≥ 75; OR: 1.81 (1.04–3.15); *p* value < 0.001. Undertriaged patients were older, more likely to be female, to have low energy trauma and to be located farther from the regional trauma centre (Table 4 – Supplement files Table 3). Glasgow coma scale of 13–15 with head injury was associated with undertriage; OR: 16.4 (6.58–41.01); *p* value < 0.001.

**Table 3:** Association between age and primary and secondary outcomes

	Univariate	P	Multivariate*	P
Primary outcome: Admission to resus room with TTA				
Admission to RR with TTA (ref age < 55)	1		1	
Age 55–74	0.53 (045–0.62)	< 0.001	0.90 (0.72–1.12)	0. 342
Age ≥ 75	0.14 (0.11–0.17)	< 0.001	0.33 (0.24–0.45)	< 0.001
Women gender (ref Men)	0.33 (0.29–0.38)	< 0.001	0.52 (0.43–0.63)	< 0.001

Secondary outcome: Admission to trauma centre	Univariate	P	Multivariate*	P
Admission to TC (ref age < 55)	1		1	
Age 55–74	0.71 (0.66–0.75)	< 0.001	0.73 (0.66–0.81)	< 0.001
Age ≥ 75	0.69 (0.66–0.72)	< 0.001	0.47 (0.42–0.53)	< 0.001
Women gender (ref Men)	0.83 (0.80–0.86)	< 0.001	0.70 (0.65–0.75)	< 0.001

Secondary outcome: Under Triage	Univariate	P	Multivariate*	P
Under triage (ref age < 55)**	1		1	
Age 55–74	2.63 (1.76–3.93)	< 0.001	1.23 (0.71–2.12)	0.458
Age ≥ 75	6.63 (4.55–9.65)	< 0.001	1.81 (1.04–3.15)	0.035
Women gender (ref Men)	2.81 (2.06–3.83)	< 0.001	1.85 (1.18–2.90)	0.007

RR: Resuscitation Room; TTA: Trauma Team Activation; TC: Trauma centre

^*^Covariate: gender, distance to trauma centre, SBP, GCS, BATT score, High energy trauma, motor vehicle accident, fall, penetrating injury (N = 36,403) ** Based on major trauma patients defined by a BATT score ≥ 8 or a MGAP < 18 (N = 690)

**Table 4:** Older injured patients (≥ 75 years) by undertriage

	Undertriaged N = 206	Non-undertriaged N = 68	*p* value
Mean age (sd)	86 (6)	83 (6)	0.002
Gender woman	137 (67)	34 (50)	0.015
High energy	46 (22)	31 (46)	< 0.001
Motor vehicle accident	13 (6)	18 (26)	< 0.001
Fall	179 (87)	45 (66)	< 0.001
Penetrating injury	9 (4)	7 (10)	0.071
SBP < 100 mmHg	54 (26)	26 (38)	0.059
GCS ≤ 8	55 (28)	45 (66)	< 0.001
GCS ≥ 13	112 (57)	14 (21)	< 0.001
Head injury	44 (65)	82 (40)	< 0.001
Distance to trauma centre	19 (13)	15 (11)	0.017
Distance to trauma centre > 20 km	204 (54)	96 (31)	< 0.001
Prehospital medical team dispatched	70 (34)	60 (88)	< 0.001
Helicopter transportation (HEMS)	39 (10)	89 (27)	< 0.001

Sd: standard deviation; SBP: systolic blood pressure; GCS: Glasgow Coma Scale; HEMS: Helicopter Emergency Medical Service

## Discussion

### Main findings

This study showed that older patients were less likely to be admitted to the regional trauma centre and to the resuscitation room with trauma team activation. Prehospital emergency physicians (MICU) were less likely to be dispatched for older patients. Older patients were less likely to receive analgesia and prehospital treatment. The risk of undertriage is increased for older patients and women, particularly for older women who have low-energy falls, head injuries, and a Glasgow Coma Scale score of 13 or higher. Geospatial analysis showed that older injured patients were more likely to be injured in urban areas and close to the regional trauma centre. Increasing distance to regional trauma centre is also a risk factor for undertriage in older patients.

The undertriage of older patients in trauma care is a major concern worldwide, as highlighted by several studies [10–12]. In the Netherlands, researchers found that 95% of undertriaged patients were aged 65 or older, with a typical profile of older patients sustaining head and/or chest trauma from falls of less than 2 m [13]. These specific trauma characteristics termed silver trauma could lead to confusion in prehospital assessment with poor recognition of high haemorrhagic risk [14]. These characteristics, which were also observed in our study, are often not adequately considered as triage criteria in triage algorithms. The challenges of accurately triaging older injured patients are compounded by the limitations of existing triage tools. The Field Triage Guidelines (FTG) used in North America to identify patients with major trauma who require transport to a trauma centre showed greater undertriage in older patients [15]. This undertriage may be due to a lack of training of paramedics who are unaware of criteria adapted to older patients [13].

Studies have shown that the inclusion of physiological criteria adapted to older patients increases sensitivity, but also decreases specificity in retrospective observational studies [16, 17]. However, Anatha et al. did not find a reduction in undertriage with specific geriatric TTA. This study estimated that 61% of older injured patients were undertriaged despite such interventions [18].

In addition, the impact of undertriage on mortality has been demonstrated, with research showing that older injured patients treated in trauma centres have lower mortality rates than those treated in non-trauma centres [19, 20].

### Clinical implication

Addressing the undertriage of older injured patients requires a comprehensive approach that includes increasing healthcare providers’ awareness of age-specific risk factors and implementing specific training programmes to improve triage decision-making. In addition, the integration of geriatric protocols into prehospital and emergency care settings can help optimise the management of older injured patients from the time of injury through to definitive care. By prioritising the needs of this growing population, healthcare systems can strive to minimise undertriage, improve patient outcomes, and enhance the overall quality of trauma care for older people. A new triage score for injured patients should be developed that takes age into account, to avoid a lack of access to trauma centres for older patients, which would increase morbidity and mortality.

### Strengths and limitations

This study has several strengths and limitations. First, we used an anonymized database containing over 40,000 patients over a 2 years study period. This inception cohort is well representative of the population without selection bias. We chose to include all injured patients requiring hospital transport as undertriaged older patients are usually not included in the database of major trauma registries. Restricting the inclusion criteria to a pre-identified major trauma population may have led to bias toward the null. Second, this wide inclusion of injured patients may have led to overestimate the undertriage as older patients were more likely to have low energy trauma compared to younger patients. However, we assessed the severity of trauma with two validated prognostic models to present adjusted results in multivariate regression analysis. We also used restriction in the study design to exclude patients without significant injury. Third, we selected covariates using a directed Acyclic Graphs (DAG) to identify appropriate covariates. We adjusted for distance to trauma centre, hospital catchment area and performed a geospatial analysis to avoid any bias due to the accident location. Distance to trauma centre is often not considered in such analyses. Fourth, we reported no missing values for the outcome and very few for exposure. We performed multiple imputation for physiological parameters to avoid any selection bias in the analysis. Fifth, we selected the worst value of physiological parameters during the prehospital intervention. Measurement error may have led to regression dilution bias and misclassification in the severity assessment of the prognostic models. Sixth, the use of prognostic score with systolic blood pressure categories of 90 mmHg for the MGAP score and 100 mmHg for the BATT score may have led to misclassification of the baseline risk for older injured patient and under-estimate of undertriage. Seventh, the results of this study are limited to the Swiss EMS setting and may be difficult to generalise.

## Conclusion

Older injured patients are at increased risk of undertriage and non-trauma team activation admission. This study identified older age, gender women, head injury without altered consciousness, greater distance to regional trauma centre as risk factor for undertriage. There is an urgent need for validated specific activation rule for trauma team in older patients. Prospective validation of specific criteria is needed.

## Supplementary Information

Additional file 1.

## Data Availability

As data was provided by the Public health service of the canton of Vaud, we are not allowed to share the data. Reasonable request could be made directly to the Public health service of the canton of Vaud.
