# Supplementary material for: Prehospital undertriage of older injured patients in western Switzerland: an observational cross-sectional study
Source: Scand J Trauma Resusc Emerg Med. 2024 Oct 8;32:100. doi: 10.1186/s13049-024-01271-5 (PMC11462677; doi:10.1186/s13049-024-01271-5)

# SUPPLEMENT FILES

sTable 1. Logistic regression model for admission to resuscitation room with trauma team activation

sTable 2. Logistic regression model for admission to trauma centre

sTable 3. Logistic regression model for undertriage

sFigure 1. Directed acyclic graph (DAG).

sTable 1. Logistic regression model for admission to resuscitation room with trauma team activation

| | OR (95% CI) | P Value |
|---|---|---|
| Age <55 years | 1 | |
| 55-74 years | 0.90 (0.72-1.12) | 0.342 |
| ≥ 75 years | 0.33 (0.24-0.45) | <0.001 |
| Women gender | 0.52 (0.43-0.62) | <0.001 |
| BATT score (by 1 point increase) | 1.09 (1.03-1.16) | 0.003 |
| SBP (by 1 point increase) linear term | 1.06 (1.03-1.08) | <0.001 |
| Quadractic term | 0.99 (0.99-0.99) | <0.001 |
| Cubic term | 1.00 (1.00-1.00) | <0.001 |
| GCS (by 1 point increase) | 0.67 (0.64-0.70) | <0.001 |
| High Energy trauma | 5.54 (1.19-6.95) | <0.001 |
| Motor vehicle accident | 1.54 (1.26-1.88) | <0.001 |
| Penetrating injury | 4.73 (3.55-6.66) | <0.001 |
| Fall | 0.59 (0.49-0.71) | <0.001 |
| Distance to trauma centre linear term | 1.08 (1.02-1.14) | 0.005 |
| Quadratic term | 0.99 (0.99-0.99) | <0.001 |
| Cubic term | 1.00 (1.00-1.00) | 0.004 |

sTable 2. Logistic regression model for admission to trauma centre

| | OR (95% CI) | P Value |
|---|---|---|
| Age <55 years | 1 | |
| 55-74 years | 0.73 (0.66-0.81) | <0.001 |
| ≥ 75 years | 0.47 (0.42-0.53) | <0.001 |
| Women gender | 0.70 (0.65-0.75) | <0.001 |
| BATT score (by 1 point increase) Linear term | 1.09 (1.03-1.16) | <0.001 |
| Quadratic term | 0.99 (0.99-0.99) | 0.001 |
| SBP (by 1 point increase) linear term | 0.99 (0.99-0.99) | <0.001 |
| GCS (by 1 point increase) | 0.75 (0.73-0.78) | <0.001 |
| High Energy trauma | 1.94 (1.70-2.23) | <0.001 |
| Motor vehicle accident | 0.82 (0.75-0.90) | <0.001 |
| Penetrating injury | 1.74 (1.33-2.27) | <0.001 |
| Fall | 0.79 (0.73-0.86) | <0.001 |
| Distance to trauma centre linear term | 0.64 (0.63-0.66) | <0.001 |
| Quadratic term | 1.01 (1.00-1.01) | <0.001 |
| Cubic term | 0.99 (0.99-0.99) | <0.001 |

sTable 3. Logistic regression model for undertriage

| | OR (95% CI) | P Value |
|---|---|---|
| Age <55 years | 1 | |
| 55-74 years | 1.23 (0.71-2.12) | 0.458 |
| ≥ 75 years | 1.81 (1.04-3.15) | 0.035 |
| Women gender | 1.85 (1.18-2.90) | 0.007 |
| Glasgow coma scale (ref GCS ≤ 8) | 1 | |
| 9-12 if Head injury | 5.27 (2.27-12.24) | <0.001 |
| 13-15 if Head injury | 16.4 (6.58-41.01) | <0.001 |
| High Energy trauma | 0.37 (0.24-0.58) | <0.001 |
| Penetrating injury | 0.34 (0.14-0.83) | <0.001 |
| Fall | 1.95 (1.22-3.11) | 0.005 |
| Distance to trauma centre linear term (for 1 Km increase) | 1.06 (1.05-1.08) | <0.001 |
| Limb trauma | 15.17 (7.10-32.41) | <0.001 |

sFigure 1. Directed acyclic graph (DAG).

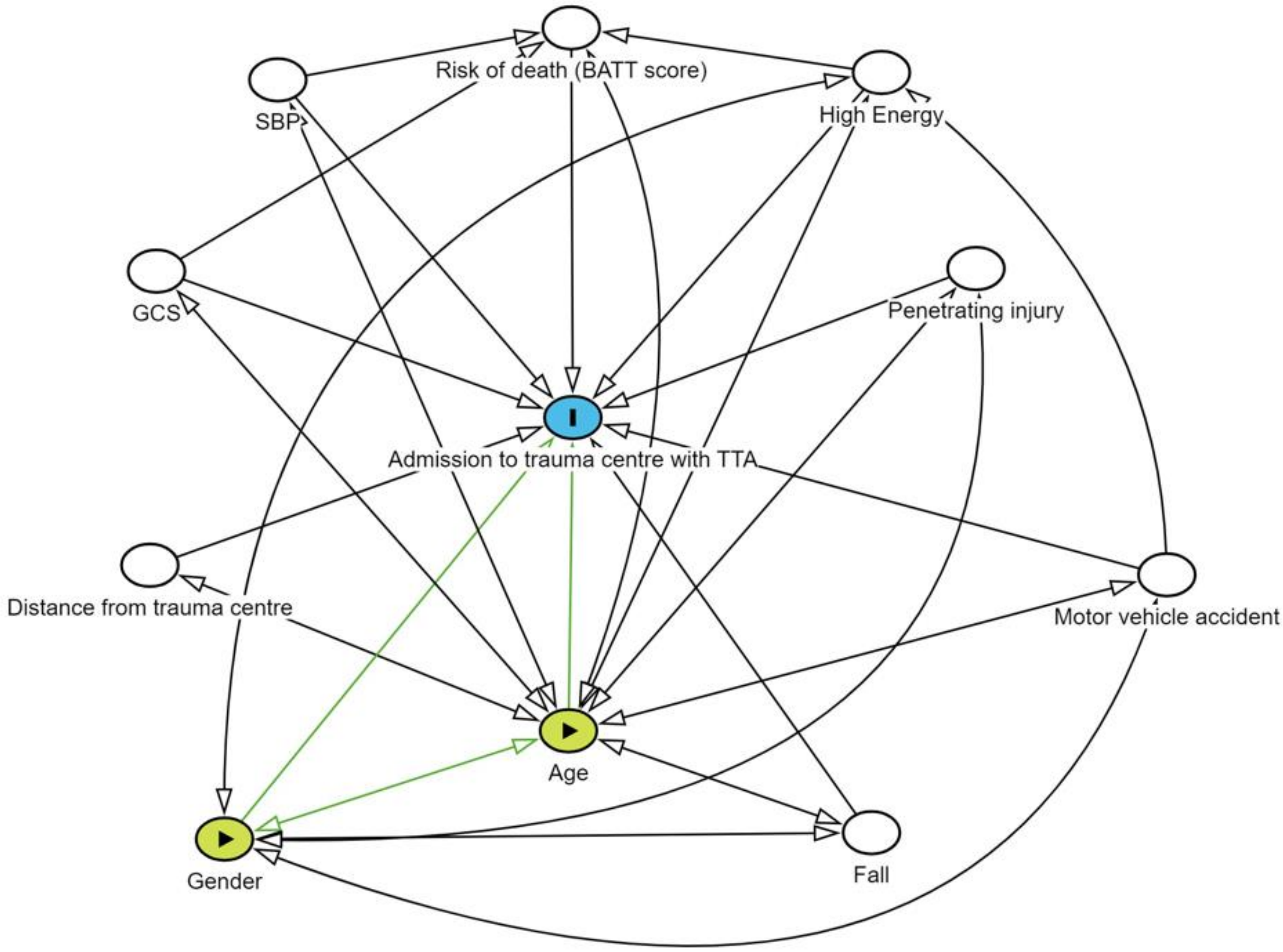

Supplement: Supplementary file 1 — Additional file 1. [file 13049_2024_1271_MOESM1_ESM.pdf]
